# Sane Approach to Optimizing the Workload in Remote Monitoring of Cardiovascular Implantable Electronic Devices

**DOI:** 10.1161/CIRCEP.124.013078

**Published:** 2025-02-20

**Authors:** Markus Sane, Toni Jäntti, Annukka Marjamaa, Elina Pennanen, Charlotte Aura, Eeva Torvinen, Leena Karjalainen, Pekka Raatikainen, Jarkko Karvonen

**Affiliations:** Helsinki University Hospital Heart and Lung Center, Finland (M.S., T.J., A.M., E.P., C.A., E.T., L.K., P.R., J.K.).; Helsinki University, Finland (M.S., T.J., A.M., J.K.).

**Keywords:** clinical relevance, heart, humans, patient safety, workload

## Abstract

**BACKGROUND:**

Remote monitoring offers an effective and safe method for monitoring patients with cardiovascular implantable electronic devices. The downside of remote monitoring is the overflow of the data. Since many of the remote monitoring transmissions are nonactionable, optimizing alert transmissions could partly overcome this problem.

**METHODS:**

We collected data on the number and the causes of all alert-, scheduled-, and patient-initiated transmissions as well as actions initiated by these transmissions in 2023. According to our strategy, all clinically nonrelevant alerts were turned off. The trend of alert transmissions and the proportion of actionable scheduled transmissions are presented. The patient safety was monitored by analyzing the premortem alerts and changes to alert settings in deceased patients, as well as the rate of actionable scheduled or patient-initiated transmissions during follow-up.

**RESULTS:**

During the study period 8182 transmissions were generated from 3732 cardiovascular implantable electronic devices. Of these, 2306 (28%) were alert transmissions, of which 57% (n=1290) were considered clinically nonrelevant. The rate of alerts decreased by 44% from January to December (0.07 versus 0.04 per device per month, *P*=0.001). Of the 3335 scheduled transmissions, 11% (364) were actionable, and the proportion of actionable scheduled transmissions remained unchanged during the follow-up period (*P*=0.08). Notably, none of the deaths were linked to the adjustment of alert settings.

**CONCLUSIONS:**

Our data indicated that active evaluation of the clinical relevance of all alert transmissions and deactivation of clinically nonrelevant alerts reduce the remote monitoring workload. However, long-term follow-up is needed to ensure that patient safety is not compromised.

**What is Known?**

- Remote monitoring of cardiovascular implantable electronic devices has been proven to be safer than standard care, yet its adoption has been lagging, for example, due to the high volume of transmissions and the consequent cost of remote monitoring.
- The majority of transmissions in remote monitoring are nonactionable but are associated with a notable workload when the findings are analyzed and documented in patient records.

**What the Study Adds**

- An approach where alert settings are actively adjusted based on the clinical relevance of the alert substantially decreases the number of nonactionable alert transmissions.
- Due to the lack of long-term data, the safety of the methodology utilized in our study cannot be absolutely assured. However, to date, we are not aware of any serious safety concerns with our approach.
- Continuous assessment of the potential benefits and shortcomings of actively adjusting the remote monitoring alert settings is important and may lead to more efficient patient care.

Remote monitoring (RM) of cardiovascular implantable electronic devices (CIEDs) has enormous potential for improving patient care. RM offers an almost real-time follow-up of the CIED function and ensures that potential malfunctions can be detected and treated in time. Unsurprisingly, RM has been shown to be safer than standard care.^1–5^ However, RM presents other challenges, with the high alert burden cited as a key barrier to its adoption.^6–8^ RM is generally regarded as cost-effective, as it decreases the number and duration of in-hospital scheduled and emergency visits, reduces the need for additional diagnostic tests, and releases physician and nurse resources for other tasks.^9,10^ The cost-effectiveness, however, depends considerably on the volume and management of RM transmissions. In clinical practice, all alerts that provide clear benefits to the patients should always be turned on to ensure patient safety, but possibly less relevant alerts could be safely turned off. A recently published expert consensus statement on the management of the remote device clinic provides guidance on appropriate alert settings and some tips for tailoring alert settings to receive only alerts that prompt clinical actions while minimizing nonactionable alerts.^11^ However, there is currently insufficient data on the impact of these suggestions on RM workload or patient safety.

The aim of this study was to assess the impact of individualizing alert settings by clinicians on the RM workload in a large CIED clinic in Helsinki, Finland. The follow-up strategy targeting the reduction of clinically nonrelevant alert transmissions has been utilized since the beginning of 2023, and here we present data supporting the recommendations outlined in the 2023 HRS/EHRA/APHRS/LAHRS expert consensus statement.^11^

## Methods

This study was approved by the institutional review board of Helsinki University Hospital. The data that support the findings of this study are available from the corresponding author upon reasonable request.

### Patient Population

The Helsinki University Hospital Heart and Lung Center is a tertiary care center with a total catchment population of 2.1 million inhabitants. It is responsible for the follow-up of all implantable cardioverter defibrillators (ICDs) and oral consent for RM cardiac resynchronization therapy devices in the Helsinki and Uusimaa hospital district (1.7 million inhabitants) and takes care of the follow-up of all patients with a conventional pacemaker living in the city of Helsinki (0.66 million inhabitants).

In our clinic, RM is considered the first option for CIED follow-up. All patients receive written and verbal information on the benefits and potential drawbacks of CIED follow-up options and provide oral consent for RM. Patients without a functioning automatic ventricular pacing threshold management algorithm and those reluctant toward RM are followed in-office. According to our alert-based RM strategy, device follow-up is performed exclusively with RM after the initial in-office visit at 1 to 3 months following device implantation. In RM, we use annual scheduled transmissions for all patients irrespective of manufacturer, and this information is evaluated and documented in the electronic patient record, as suggested in the recent consensus statement.^11^ In addition, patient-initiated transmissions are available to all patients with devices capable of this function, and the transmissions are at the patient’s discretion. In our practice, patients are instructed to contact RM staff if any symptoms attributable to the CIED have emerged, and often the first step is to request a patient-initiated transmission to evaluate the CIED function.

At the end of the study period, 3732 patients with CIED were under RM at our institute. The number of patients in RM is collected at the end of each month, and this number was used as the denominator when the monthly alert rate per device was calculated. Over the course of the study, the number of patients in RM increased by 7%.

The distribution of device types and manufacturers is summarized in Table 1. It is noteworthy that insertable loop recorders were excluded from this analysis.

**Table 1.**
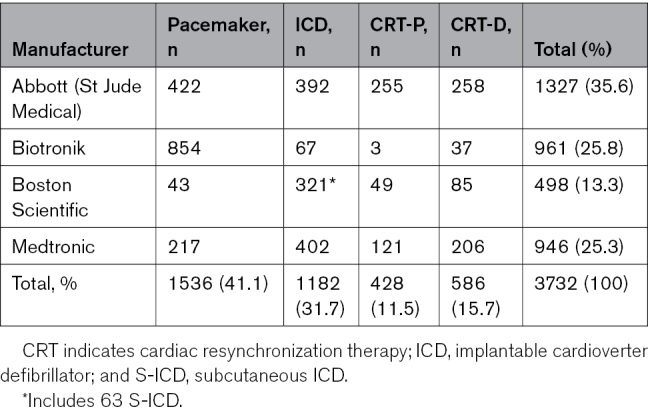
Distribution of Different Devices in Remote Monitoring

### Alert Settings

The alert settings in devices and RM portals vary depending on the device type, generation, and manufacturer. In our practice, all lead-related alerts (sensitivity, threshold, and impedance) have been turned on, if possible, by utilizing manufacturer-specific nominal values. In all patients, the alerts for elective replacement indicators and, for ICD/cardiac resynchronization therapy-D patients, antitachycardia pacing and shock therapy are turned on. Our practice is in line with the TRUST study (The Lumos-T Safely Reduces Routine Office Device Follow-up) core set, with minor differences are related to longer atrial fibrillation (AF) burden and device transmission failure of 3 weeks.^12^ Otherwise, the alert settings are tailored based on clinical criteria to minimize nonactionable alerts and only receive alerts that prompt actions. For example, of the clinically relevant and nonrelevant alerts, as well as alerts with uncertain clinical relevance, are presented in Table 2.

**Table 2.**
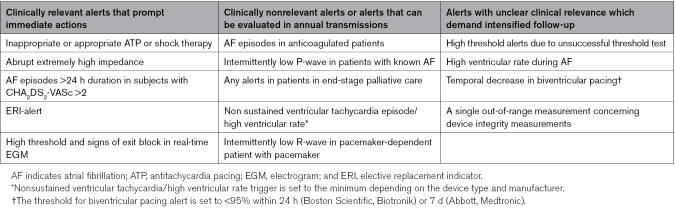
For Example, of Different Types of Alert Transmissions Based on Helsinki University Hospital Heart and Lung Center Clinical Practice

**Figure 1.**
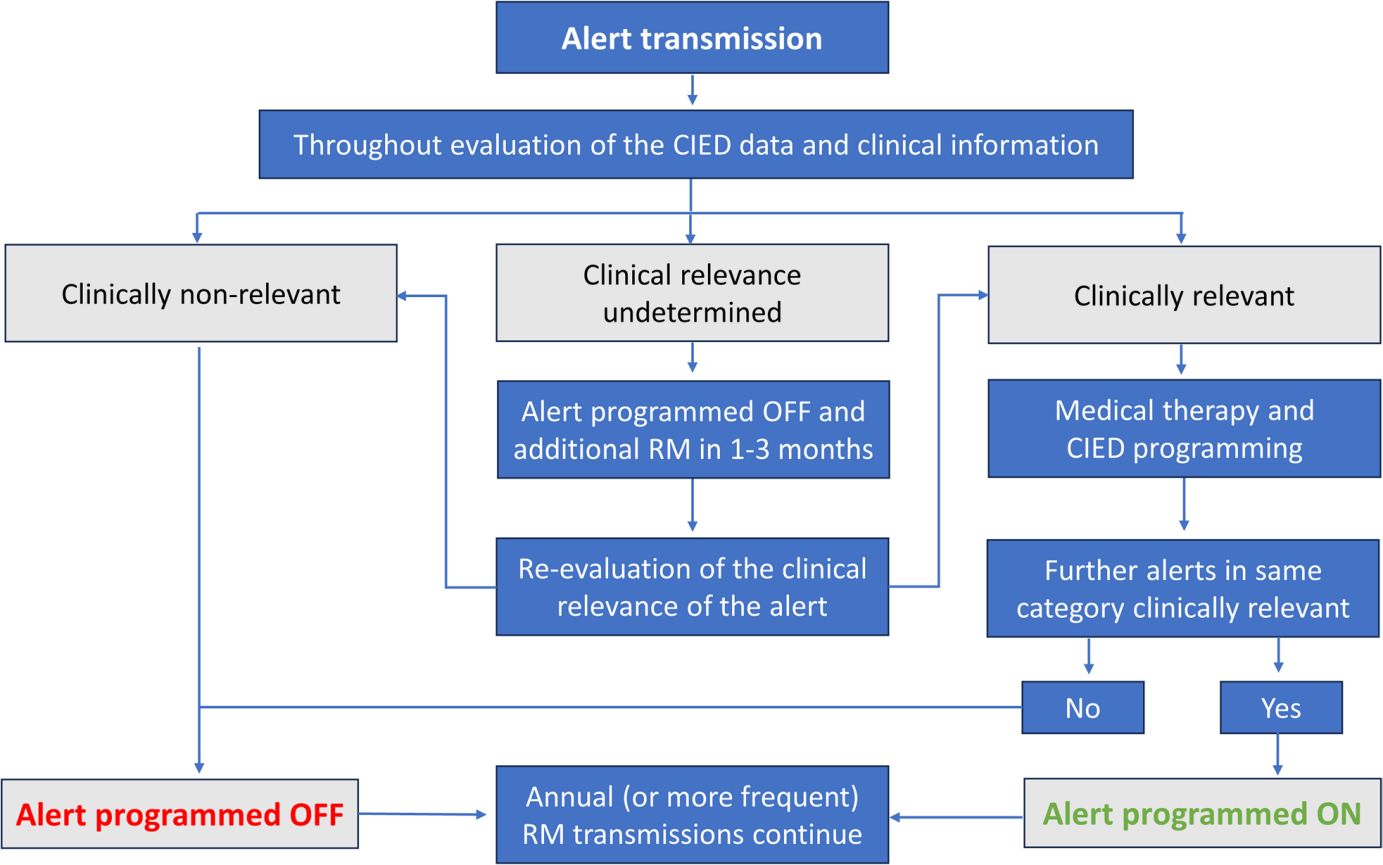
**An approach to adjust the alert settings based on the clinical relevance of the alert transmissions.** CIED indicates cardiovascular implantable electronic device; and RM, remote monitoring.

Since the beginning of 2023, explicit instructions have been in place to systematically assess all alert transmissions (Figure 1). In brief, clinicians have been advised to deactivate clinically nonrelevant alerts and reevaluate alerts with undetermined clinical relevance within 1 to 3 months by utilizing repeat transmission (additional scheduled transmission). In addition to the episodes with undetermined clinical relevance, it was instructed to program an additional scheduled transmission for all lead-related alerts, high ventricular rate (HVR) during AF, and low percentage of biventricular pacing. The additional transmissions are evaluated by the same electrophysiologist who was responsible for the evaluation of the original alert.

At the initiation of RM, the alert for AF episodes is turned off for anticoagulated patients with known AF. In other patients, the threshold for the AF burden alert is set to 6 hours irrespective of the CHA_2_DS_2_-VASc score, and the threshold for the average ventricular rate alert during an AF episode to is set >100 beats per minute for a 12-hour (Abbott) or 24-hour period (Biotronik and Medtronic). Shorter AF episodes or AF episodes in anticoagulated patients do not trigger transmission, but all the data are stored in the RM portals, enabling evaluation of the episodes during annual transmissions.

In our practice, the nonsustained ventricular tachycardia (NSVT) and HVR episodes are evaluated only using the annual scheduled transmissions. That is, the alerts for NSVT/HVR have been switched off from the RM portals at the time of implantation since 2022, and in patients with a CIED implanted before that, the trigger is turned off after the first NSVT/HVR alert. Also, the ventricular pacing percentage alert is nominally turned off, and the potential changes in pacing are evaluated from the annual transmissions.

The threshold for the biventricular pacing alert is set to <95% within 24 hours (Boston Scientific, Biotronik) or 7 days (Abbott, Medtronic).

### Workflow of RM Transmissions

Our RM clinic staff includes 4 trained nurses and 6 electrophysiologists. On average daily, three and a half nurses work full-time on RM and half of an electrophysiologist’s working day is dedicated to RM. Every morning, the nurses carefully scan all 4 RM portals, and all alert transmissions are evaluated on the same business day, regardless of the alert urgency category. Alerts for the antitachycardia pacing or shock therapy, as well as additional scheduled transmissions due to alerts with unclear clinical significance, are referred to the electrophysiologist. Nurses primarily evaluate other alert transmissions, utilizing the guidance for various alerts provided in Figure S1. They consult the electrophysiologist as necessary. The nurses proactively integrate pertinent information from RM portals, such as electrogram or trend curves, into the electronic patient record system, saving time for the electrophysiologist by eliminating the need to log into RM portals for consultations.

The scheduled and patient-initiated transmissions are primarily evaluated by nurses under the supervision of the electrophysiologist in charge of RM. Patient-initiated transmissions undergo evaluation on the following business day, while annual scheduled transmissions are assessed within 1 to 2 weeks. Due to the shortage of workforce, the analysis of scheduled transmissions was delayed in April and postponed to May.

### Data Collection

The primary cause for individual transmissions and the actions taken were systematically collected using a standardized data sheet (Microsoft Excel) by the nurses responsible for RM (Table S1). In brief, the transmissions were classified according to the type of the transmission, manufacturer, device type and serial number, need and cause for electrophysiologist or manufacturer representative consultation, cause and action for alert transmissions or clinically relevant changes identified in scheduled or patient-initiated transmissions. In addition, the information on whether the same alert had recurred within a period of <1 week, 1 month, 3 months, or 6 months was collected. If the alert had not occurred in the preceding 6 months, it was classified as a new alert. Starting from May 1, 2023, a distinction was made between additional scheduled and annual scheduled transmissions. Overall, the data were collected from the year 2023.

The specific data for the total number and duration of atrial mode switch episodes consequently triggering atrial high rate episode alert and NSVT/HVR episodes stored in the CIED memory were collected from the Merlin.net RM portal with the assistance of Abbott RM service. Abbott CIEDs were chosen due to previous collaboration and because in our RM, Abbott is largest manufacturer and different CIEDs types are comprehensively represented.

The number of patients who died during the study while enrolled in RM and the data regarding changes in alert settings for these patients were collected separately. The patient-level data from electronic patient records system were analyzed from all deceased patients and patients with alert or additional scheduled transmissions because of lead integrity alert or NSVT/HVR episode in any transmission leading to an in-office follow-up. In addition, the electronic patient records were analyzed from the 20 subjects with largest number of alerts transmissions during the follow-up. The RM information of all deceased patients can be preserved in the RM portals for a period of 3 to 7-years if a patient is deactivated from the RM. This practice was initiated in the beginning of 2023.

The adverse effects related to RM were also evaluated by analyzing all the HAIPRO events related to pacemakers. A HAIPRO is a web-based reporting tool for patient safety incidents and a notification is always made when an event or near miss causing harm to the patient has been detected.^13^

### Statistics

The data were analyzed with IBM SPSS 25.0 for Windows (IBM Corp, Armonk, NY). Linear regression was used to evaluate the statistical significance of changes in the monthly rate of alert transmissions or new alert transmissions, as well as the proportion of nonactionable alert transmissions and alerts leading to in-office follow-up. The comparison of alert transmissions is presented between January and December 2023.

The change in the proportion of actionable scheduled transmissions was also analyzed with linear regression, but the analysis was conducted from May to December due to the distinction made between additional scheduled and scheduled transmissions after first of May.

The difference between actionable scheduled transmissions in patients with or without alert transmissions in the first half of the study was analyzed with the χ^2^ test. Statistical significance was considered at the 5% level.

## Results

**Figure 2.**
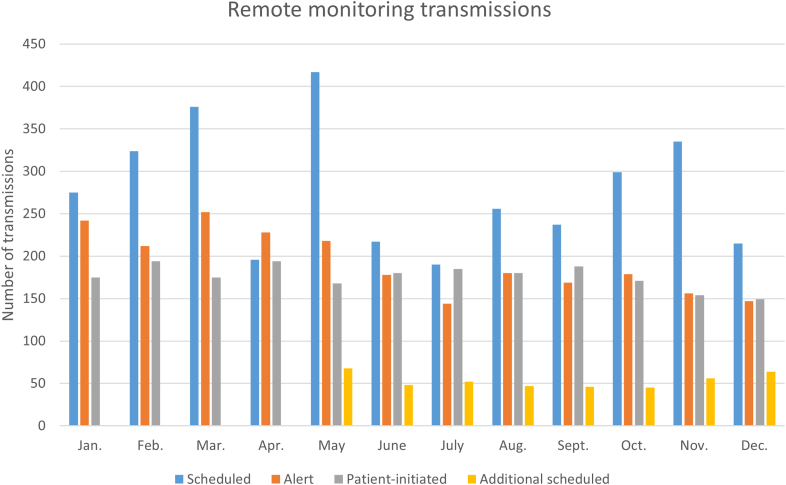
The number of remote monitoring transmissions during the follow-up.

In total, 8182 transmissions were generated from 3732 CIEDs. Among these, 2306 (28.2%) were alert transmissions, 3335 (40.8%) were scheduled transmissions, 426 (5.2%) were additional scheduled transmissions, and 2113 (25.8%) were patient-initiated transmissions. This corresponds to an average monthly transmission rate per device of 0.2 (0.05 for alert, 0.08 for scheduled, 0.01 for additional scheduled, and 0.05 for patient-initiated transmissions; Figure 2).

### Alert Transmissions

**Figure 3.**
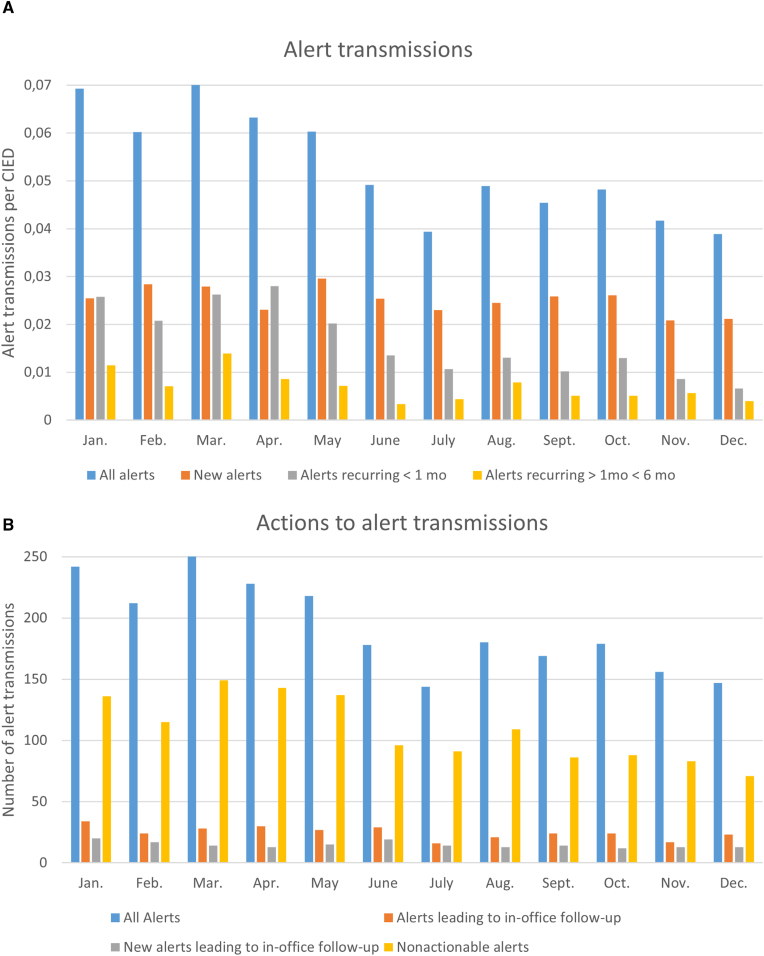
The trend of alert transmissions and actions taken during the follow-up.

The rate of alert transmissions decreased by 44% from January to December, from 0.07 to 0.04 per device per month (Figure 3A; *P*=0.001). The mean number of new alerts per device was 0.025 per month, and the rate decreased by 17%, from 0.025 to 0.021 (*P*=0.04). The change in alert rate per device type (pacemaker, ICD, and cardiac resynchronization therapy) is presented in Figure S2. The rate of alerts recurring within <1 month and 1 month to <6 months decreased by 75% and 65%, respectively (0.025 versus 0.007 per month; 0.01 versus 0.004 per month (*P*=0.001, *P*=0.001, respectively).

The number of nonactionable alerts decreased by 48%, from 136 to 71 (*P*=0.001), whereas the proportion of alerts leading to in-office follow-up or guidance to seek care at the emergency department remained unchanged (*P*=0.8; Figure 3B). Of all the alerts that necessitated an in-office visit or guidance to seek care at the emergency department, 60% (177/296) were new alerts. The most common causes and actions taken after alert transmissions are listed in Table 3.

**Table 3.**
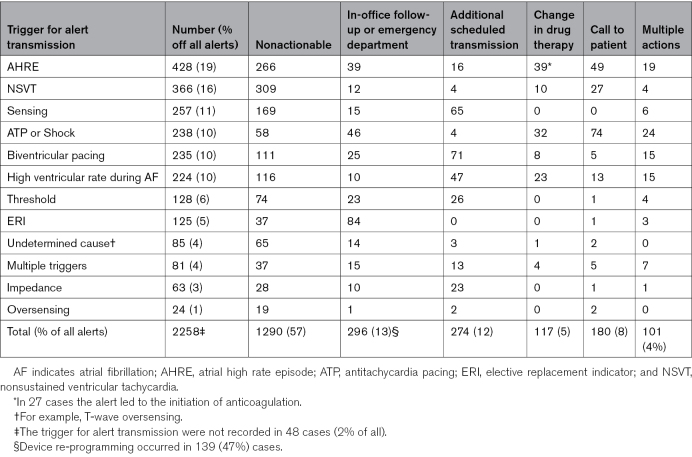
The Most Common Causes and Actions Taken After alert Transmissions

In total, alert transmissions were generated from 1124 (30%) individual patients, and 476 (13%) patients generated >2 alerts, while 88 (2%) patients generated >5 alerts, corresponding to 30% of all alerts. The causes for multiple alerts in the same subject were mainly due to antitachycardia pacing, monitored ventricular tachycardia episodes, T-wave oversensing, or lack of adherence to the instructions concerning the management of alert transmissions for an unknown cause.

### Additional Scheduled Transmissions Due to Alerts or Findings from Scheduled Transmissions of Undetermined Relevance

A total of 426 additional scheduled transmissions were evaluated, of which 274 (64%), 93 (22%), and 59 (14%) were programmed due to alert transmission, findings from an annual scheduled transmission, or previous additional scheduled transmissions, respectively. The most common causes for additional scheduled transmissions were the evaluation of biventricular pacing (24%, n=106), HVR during AF or AF episode (17%, n=73), threshold (11%, n=47), sensing (8%, n=33), and impedance (6%, n=26).

The additional scheduled transmissions led to in-office follow-up in 35 patients (11% of all additional transmissions).

### Scheduled Transmissions

**Figure 4.**
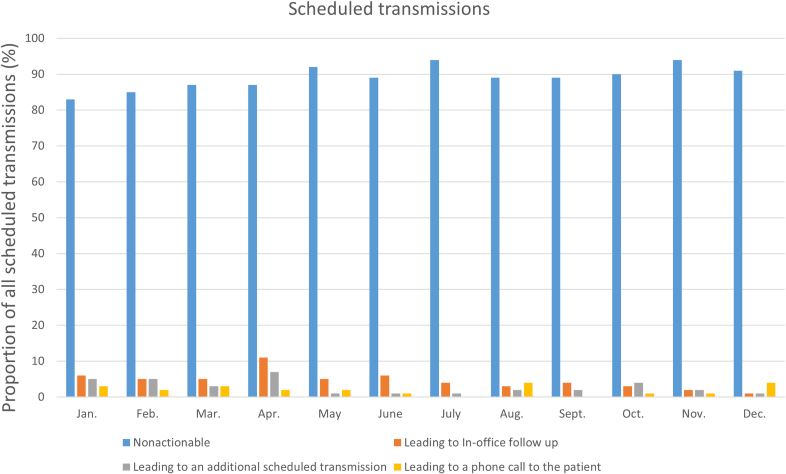
The trend of the proportion of different actions taken after scheduled transmissions during the follow-up.

In total, 3335 scheduled transmissions were reviewed. Among all scheduled transmissions, 89% (n=2971) were nonactionable. On average, 4.5%, 3%, 2%, and 0.6% led to in-office follow-up, additional scheduled transmission, a phone call to the patient, or changes to medication, respectively. The number and proportion of actionable scheduled transmissions remained unchanged during the study period (*P*=0.08; Figure 4). The proportion of actionable scheduled transmissions was equal in the 170 patients who had at least 1 alert transmission in the first half of the follow-up and scheduled transmissions in the last half compared with patients who has no alert transmissions during the first half and scheduled transmissions in the last half (92% versus 91%; *P*=0.2).

### Additional Information

The overall number of NSVT/HVR episodes stored in the CIEDs of Abbott was 5687 and the episodes were triggered, from 387 individual patients (29% of all patients with Abbott CIEDs).

The number of atrial high-rate episodes >6 minutes was 1329, >1 hour 810, >6 hours 439 >24 hours 175.

In our RM cohort, the total number of deceased patients during the study period was 173, with a mean age of 79 years. Only 18% (n=31) of the deceased patients generated an RM alert within the last 6 months before the time of death. Among 25 of them, the alert settings were modified before demise because the alerts were deemed clinically nonrelevant, as the patients were in end-stage palliative care. The causes of the alerts were: HVR during AF (9, 36%), low P-wave during AF (4, 16%), decreased biventricular pacing (3, 12%), AF episode in anticoagulated patient (2, 8%), ICD therapy disabled (2, 8%), unsuccessful threshold test (2, 8%), NSVT (1, 4%), low R-wave (1, 4%) and atrial impedance (1, 4%). Overall, none of the deaths were linked to the adjustment of alert settings.

In total, 55 HAIPRO reports were registered in 2023 in the Helsinki University hospital Heart and Lung Center. Of these, 3 were related to RM of CIEDs (Table 4), but none were associated with the adjustment of alert settings.

**Table 4.**
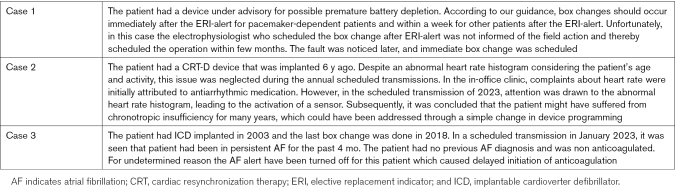
The Details of HAIPRO Events Related to Remote Monitoring

### Nurse and Electrophysiologist Collaboration

Overall, nurses evaluated 1118 (49%), 3256 (98%), and 2041 (97%) of all alerts, scheduled, and patient-initiated transmissions, respectively. The need for electrophysiologist consultation occurred after 283 (25%), 271 (8%), and 140 (7%) of alert, scheduled, and patient-initiated transmissions.

## Discussion

This study presents the results on how RM workload can be reduced by actively adjusting alert settings based on the clinical relevance of the alerts. Our approach could be described as pragmatic, involving the optimization of RM settings at initiation, efficient interplay between the RM nurses and electrophysiologists, and continuous refinement of alert settings. Within a year, our approach led to a significant reduction in the volume of alert transmissions. As the workload of RM mainly consists of analyzing the transmissions and documenting the findings in patient records, the number of transmissions directly impacts the workload. Based on our findings, we believe that an approach aimed at reducing clinically nonrelevant transmissions results in more efficient RM.

It has been shown that the majority of RM alert transmissions are nonactionable.^6,14^ The latest consensus statement from HRS/EHRA/APHRS/LAHRS addresses RM alerts and recommends adjusting or deactivating clinically nonrelevant alerts, such as AF episodes in anticoagulated patients or other clinical events that have already been addressed.^11^ The challenge remains in accurately identifying these alerts and the consensus statement does not present specific guidance for alerts such as a single high or low lead integrity measurement value. In our earlier study,^15^ we showed that a significant proportion of the threshold, impedance, or sensing alerts recurred shortly after being addressed. Consequently, we initiated an approach where lead integrity alerts without severe features received adequate follow-up, with evaluation subsequently conducted by the same electrophysiologist. This method was deemed preferable to leaving alert settings unchanged and waiting for a recurrence. In the latter scenario, evaluations might be conducted by a different physician, and previous changes might not be thoroughly noted, especially amid a deluge of other alerts. This approach is also advocated in a recent review of lead failures, which emphasizes that any single unclear out-of-range impedance measurement should prompt intensified follow-up.^16^ Our approach aligns with the recent consensus statement^11^ and the results are encouraging. Overall, during the follow-up, the rate of alerts decreased by 44%, and at the end of the follow-up period, the monthly rate of alerts per device was markedly lower (0.04 versus 0.16) than in the recent report by O’Shea et al^6^ analyzing the transmissions from >26 000 CIEDs. Notably, many patients in our cohort had no RM alert transmissions during the follow-up, which aligns with the results of the TRUST study, where half of the patients did not transmit any alerts during the study.^14^ However, a certain monthly base rate of new alerts, averaging 0.03 transmissions per device, can be anticipated, with the majority leading to immediate actions.

The programming of the RM alert settings during the initiation of RM also impacts RM workload. The HRS/EHRA/APHRS/LAHRS consensus statement highlights that, ideally, the optimization of the alert settings should occur at the time of implantation based on individual clinical circumstances.^11^ It recommends that NSVT/HVR alerts should be utilized in selected ICD and patients with pacemaker. Discrepancies exist in the outcomes related to NSVT/HVR episodes in patients with CIEDs, in some studies NSVT/HVR episodes were not associated with impaired prognosis^17,18^ as opposed to studies where NSVT episodes were associated with an increased risk of heart failure hospitalization or with the risk of appropriate therapy.^19,20^ A thorough action after NSVT alert might prevent the risk of adverse events but to our knowledge, no prior study has addressed this matter. On the other hand, Ploux et al^21^ recently proposed that NSVT/HVR alerts caused by ventricular oversensing may serve as an early and sensitive indicator of lead failure. Our data illustrate that the possibility of earlier diagnosis of lead failure or the prevention of adverse events associated with NSVT is related to a substantial workload. In our cohort of patients with a CIED from Abbott, one-third (387/1327) had at least 1 NSVT/HVR episode, and nearly 1 in10 (n=127) had > 5 NSVT/HVR episodes per year, with a total of >4 NSVT/HVR alerts anticipated per patient annually. It should be highlighted that in our practice NSVT/HVR episodes are evaluated during annual transmissions, and if an NSVT/HVR episode is caused by oversensing or >1 episode per month is observed (Supplemental Material), the nurse consults the electrophysiologist for possible actions. Nevertheless, this approach is subject to questioning, and in the future, artificial intelligence may be able to assist in evaluating NSVT/HVR alerts,^22^ potentially overcoming the challenges associated with these alerts.

The consensus statement does not give any specific instructions with regards to the AF burden in nonanticoagulated patients,^11^ and the optimal threshold for AF alerts remains a subject of debate. The recent landmark studies assessing the benefits of oral anticoagulation after subclinical AF (SCAF) showed modest benefits of oral anticoagulation.^23,24^ Interestingly, in the subgroup analysis of the ARTESiA trial, it was observed that the duration of SCAF (<24 hours) or the frequency of SCAF episodes were not associated with the stroke risk, but the benefit of oral anticoagulation was evident in patients with a CHA_2_DS_2_-VASc score >4.^25,26^

In our practice, we have estimated that an alert threshold of 6 hours for SCAF offers the best compromise, as the shorter the threshold is the higher is the likelihood of false positive findings.^27^ We consider that shorter episodes (<6 hours) can be safely evaluated in annual scheduled transmissions, but in the light of the recent findings from the ARTESiA trial our practice may need to be refined. On the other hand, it was also observed that the stroke risk was lowest among patients in whom no SCAF was detected in the past 6 months before enrollment, indicating that, in certain cases, longer follow-up could be beneficial before the decision of life-long oral anticoagulation.^26^

It should be noted that device-detected AF episodes have been shown to predict the risk of heart failure hospitalization, and turning off AF alerts in anticoagulated patients may delay the restoration of sinus rhythm.^28^ Therefore, it is possible that AF alerts might be clinically valuable also to anticoagulated patients to guide rhythm control, but further study is needed. If AF alerts are turned off, we advise that annual scheduled transmissions should be utilized to evaluate AF burden and whether additional measures for rhythm control should be taken, especially in heart failure patients.

## Strengths and Limitations

The strengths of this study include a large sample size and detailed data regarding actions on RM transmissions. Our strategy has the potential to increase the adoption of the RM, as it allows for reevaluation of the workload and staffing requirements. Currently, in our clinic 3.5 nurses are responsible for RM of almost 4000 patients with CIEDs. This is markedly less than the proposed minimum of 3 full-time equivalents per 1000 devices^11^ suggesting that our approach could reduce the costs of RM which has also been cited as a factor for lagging adoption.^29^

It should be borne in mind that this was a nonrandomized observational study, and we evaluated patient safety, the most important aspect of CIED therapy indirectly. Due to the limited resources, we were unable to evaluate patient safety by analyzing individual electronic patient records for all monitored patients. Therefore, we are unable to ensure that our approach does not lead to hospitalizations for heart failure or atrial arrhythmias that might have been prevented with earlier arrhythmia or heart failure detection.

Modern CIEDs are considered extremely reliable,^30^ and it is unlikely that an adequately powered randomized study evaluating the optimal RM alert settings and patient safety will ever be conducted. We evaluated the safety aspect diversely by assessing patient-level data from all deceased patients and patients with lead integrity alerts or NSVT/HVR episodes leading to in-office follow-up. We found that alert adjustments was not associated with the deaths, and there were no signs of harm due to delayed detection of lead failure or NSVT/HVR episodes with our approach. Secondly, we observed that the proportion of clinically actionable scheduled transmissions remained unchanged during the follow-up period and the rate and proportion of patient-initiated transmissions resulting in in-office visits were extremely low (3%, 60/2113) and comparable to the previous data from the year 2022.^15^ Therefore, it appears that turning off seemingly nonrelevant RM alarms after thorough evaluation does neither delay reaction to the same issue in subsequent scheduled transmissions nor cause a shift from automatic alert transmissions to symptom-driven patient-initiated transmissions. Finally, none of the HAIPRO cases related to RM were due to adjustment of alert settings. Although the HAIPRO data has not been scientifically validated its coverage for patient safety issues is good and >22 000 HAIPRO cases are reported annually in Helsinki and Uusimaa hospital district. However, the follow-up period was relatively short and thus, the long-term safety of our approach with regards to lead failure detection, decompensation of heart failure due to low biventricular pacing or development of persistent AF, or delayed intensification of antiarrhythmic therapy for ICD patients with multiple NSVT episodes remains to be established. Therefore, we believe that continuous assessment of the potential benefits and shortcomings of actively adjusting the RM alert settings is important and may lead to more efficient patient care.

## Conclusions

RM ensures effective and almost real-time follow-up of patients with CIED but is associated with data deluge. We demonstrated that active optimization of the RM alert settings has the potential to improve the efficacy and cost-effectiveness of patient with CIED care by reducing the rate of clinically nonrelevant alerts. However, more data on the long-term safety of this approach is needed.

## Article Information

### Sources of Funding

This study was funded by grants from the Aarno Koskelo foundation, the Paavo Nurmi foundation, the Päivikki and Sakari Sohlberg foundation, the Finnish Foundation for Cardiovascular Research, and Heart and Lung Center Y-grants.

### Disclosures

None.

### Supplemental Material

Supplemental Methods

Table S1

Figures S1 and S2
